# Treatment-Related Coronary Disorders of Fluoropyrimidine Administration: A Systematic Review and Meta-Analysis

**DOI:** 10.3389/fphar.2022.885699

**Published:** 2022-05-13

**Authors:** Yajie Lu, Shizhou Deng, Qiongyi Dou, Wei Pan, Qingqing Liu, Hongchen Ji, Xiaowen Wang, Hong-Mei Zhang

**Affiliations:** Department of Clinical Oncology, Xijing Hospital, Air Force Medical University of PLA, Xi’an, China

**Keywords:** coronary disorder, 5-FU, capecitabine, meta-analysis, fluoropyrimidine

## Abstract

**Background:** Coronary disorders are recognized as the most common manifestation of fluoropyrimidine-related cardiotoxicity in clinical practice. However, there are limited and conflicting data on the incidence and profiles of fluoropyrimidine-related coronary disorders. In this meta-analysis, we aimed to systematically assess the incidence of all-grade and grade 3 or higher fluoropyrimidine-related coronary disorders, and further explore the factors that influence its occurrence.

**Methods:** Studies reporting the fluoropyrimidine-related coronary disorders were retrieved from a systematic search of English literature in the PubMed, Web of Science, Medline, and Cochrane database from 1 Jan 2001, to 1 Jan 2022. The NIH assessment tool was used to evaluate the quality of each study. The data of basic study characteristics, treatment details, and results of coronary toxicities were extracted. According to the results of the heterogeneity test (I^2^ and *p*-value statistic), a random-effect model or fixed-effect model was selected for the pooled analysis of the incidence of adverse coronary events. Subgroup analysis was conducted to further explore the risks influencing the occurrence of fluoropyrimidine-related coronary disorders. The stability and publication bias of our results were evaluated by sensitivity analysis and Egger test, respectively.

**Results:** A total of 63 studies were finally included in our pooled analysis, involving 25,577 patients. The pooled cumulative incidence of all-grade and grade 3 or higher coronary disorders was 2.75% (95% CI 1.89%–3.76%) and 1.00% (95% CI 0.62%–1.47%), respectively. The coronary disorders were most reported as myocardial ischemia (1.28%, 95% CI 0.42%–2.49%) and angina/chest pain (1.1%, 95% CI 0.54%–1.81%). Subgroup analysis revealed that studies in the female-only population seemed to have a lower incidence of fluoropyrimidine-related coronary disorders. The occurrence of adverse coronary events varied among different tumor types. Patients with esophageal cancer have the highest coronary toxicity (6.32%), while those with breast cancer have a relatively lower incidence (0.5%). Coronary disorders induced by 5-FU monotherapy are more frequent than that induced by capecitabine (3.31% vs. 1.21%, *p* < 0.01). Fluoropyrimidine combination therapy, whether combined with other chemotherapy drugs, targeted therapy drugs, or radiotherapy, significantly increased the incidence of coronary complications (*p* < 0.01).

**Conclusion:** This meta-analysis has defined the incidence of fluoropyrimidine-related coronary disorders and depicted its epidemiological profiles for the first time, which may provide a reference for clinical practice in cancer management.

## Introduction

With the continuous development of chemotherapy, radiotherapy, and new treatment technologies, the survival of cancer patients has been greatly improved. Meanwhile, the cardiovascular toxicity related to anti-tumor therapy has become increasingly prominent, which is one of the important causes of death due to treatment-related complications (Curigliano et al., 2016). Cardio-Oncology, an emerging interdisciplinary field, focuses on cardiovascular disease in cancer patients, and has developed rapidly in recent years (Koutsoukis et al., 2018). The incidence and spectrum of cardiotoxicity vary widely by chemotherapeutic regimens. The cardiotoxicity of anthracyclines has been extensively studied and highly concerned over the past 2 decades (Lotrionte et al., 2013; Smith et al., 2010). However, fluoropyrimidine (5-fluorouracil (5-FU), capecitabine, S-1, Tas102, etc.) induced cardiotoxicity has not been attracted equal attention.

The coronary disorder is one of the typical adverse reactions induced by chemotherapy agents, such as 5-FU and capecitabine, which often refers to the transient contraction of coronary artery and thrombus formation, causing varying degrees of myocardial ischemia, and resulting in the clinical syndrome of angina pectoris, myocardial infarction, even sudden death (More et al., 2021). Chest pain with typical or atypical angina pectoris is the most prominent manifestation of the coronary disorder, which has directly been visualized during coronary angiography (Baldeo et al., 2018; Das et al., 2019; Gao et al., 2019).

Despite some studies that have focused on fluoropyrimidine-induced coronary disorder, most of them were conducted with small samples or just case reports (Karakulak et al., 2016; Ben-Yakov et al., 2017; Sedhom et al., 2017). The reported incidence of fluoropyrimidine-related coronary disorder varies from 0% to 35% (Pai and Nahata, 2000; Sara et al., 2018; Lestuzzi et al., 2020), which is a too wide range to provide valuable reference for clinical practice. In addition, some studies suggested that the occurrence of coronary disorder depended on the different fluoropyrimidine drugs, route of administrations, dosage schedules, and co-administered agents (Depetris et al., 2018; Kanduri et al., 2019). However, there is no consensus on the incidence, profiles, and risk factors of fluoropyrimidine-related coronary disorders. An accurate description of the incidence and epidemiological characteristics of coronary vasospasm is the basis for guiding clinical practice and is very crucial for the early identification and prevention of ischemic events caused by fluoropyrimidines. Obviously, the currently available data are not yet sufficient for drawing definite conclusions. Therefore, in this systematic review and meta-analysis, we are dedicated to comprehensively and systematically evaluating the incidence and epidemiological characteristics of fluoropyrimidine-induced coronary disorders and to further exploring the factors influencing its occurrence using a method of single-rate meta-analysis.

## Materials and Methods

### The Definition of Coronary Disorder

The coronary disorder of interest in this study was defined as a group of symptoms represented by chest pain syndrome, including angina pectoris, myocardial ischemia, myocardial infarction, and acute coronary syndrome. The fluoropyrimidine-related coronary disorders were recognized by the new occurrence of a chest pain at rest in the presence of recent fluoropyrimidine administration with or without electrocardiogram (ECG) or biomarker changes.

### Search Strategy and Selection Criteria

Literature search and study selection were conducted under the PRISMA guidelines. Studies reporting the fluoropyrimidine-related coronary disorders were retrieved from a systematic search of English literature in the PubMed, Web of Science, Medline, and Cochrane database from 1 Jan 2001 to 1 Jan 2022. The search strategy was determined after several pre-retrievals and finally combined the following two sorts of items: 1) “fluoropyrimidine” OR “5-FU” OR “capecitabine” OR “S-1” OR “Tas102”; 2) “cardiotoxicity” OR “coronary vasospasm” OR “chest pain” OR “angina” OR “myocardial ischemia” OR “myocardial infarction” OR “acute coronary syndrome.” Studies had to meet the following inclusion criteria: 1) patients with a diagnosis of solid malignances; 2) articles explicitly reported the coronary disorders as defined above, and it is associated with fluorouracil-containing treatment; 3) the sample size was greater than 20; 4) the full-text was available; 5) prospective or retrospective clinical studies. Reviews, letters, comments, case report, meeting abstract were excluded.

### Methodological Quality Assessment and Data Extraction

The quality of included studies was assessed using the quality assessment tool of the National Institutes of Health (NIH) (Nhlbi Study Quality Assessment Tools, 2020, Supplementary Table S1). The reviewers could select “YES,” “NO,” or “Cannot Determine/Not Applicable/Not Reported” for each item in the list. Based on their responses, the quality of each study was graded as “good,” “fair,” or “poor.” The incidences of fluoropyrimidine-related coronary disorders of all-grade and grade 3 or higher were the main outcomes in this meta-analysis. The data of basic characteristics (first-author, publication year, study design, country or region, age, gender, tumor type, and sample size), treatment details (treatment type, line, regimen, and dosage), and the incidence of fluoropyrimidine-related coronary disorders were extracted and documented. Two authors (Lu and Deng) independently searched the literature, assessed the quality of included studies, and extracted and cross-checked the data.

### Statistical Analysis

The incidence of fluoropyrimidine-related coronary disorders in each study was shown as a percentage calculated using a division method ( $\frac{the\;sum\;of\;cornary\;disorder}{the\;sum\;of\;total\;patients}\times 100\%$ ). The Cochran’s chi-squared test reporting I^2^ statistic and *p*-value was used to test heterogeneity, and if heterogeneity exists (I^2^ > 50% or *p* < 0.1), a random-effect model was conducted, otherwise, a fixed-effect model was adopted. The pooled incidence was achieved by a single rate meta-analysis method, shown as a proportion and 95 confidence intervals (CI). Subgroup analyses were performed based on study-level characteristics (e.g., publication period, study design, gender, age, tumor, treatment type, regimen, and so on) for all-grade and grade 3 or higher adverse coronary events. Sensitivity analyses were conducted to evaluate the stability of our results. Publication bias was shown by funnel plot symmetry and statistically checked using the Egger test. For all tests, *p*-values less than 0.05 were considered statistically significant. All the statistical process of this meta-analysis was performed using R software (version 4.0.6, MathSoft, Massachusetts) with “meta,” “rmeta,” and “metafor” packages.

## Results

### Eligible Studies and Characteristics

**FIGURE 1:**
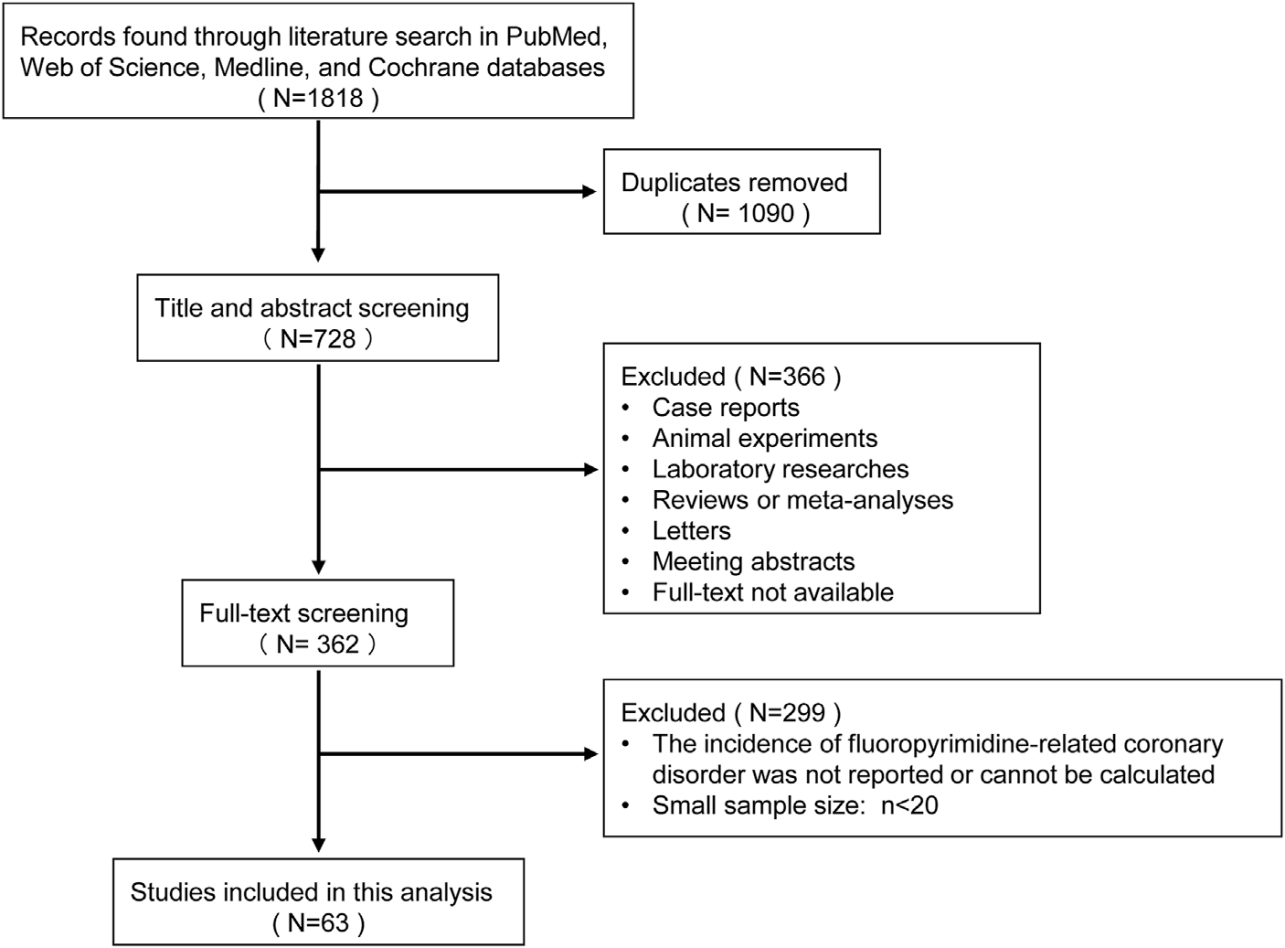
The flow diagram for literature selection, screening, and inclusion.

A total of 1818 initial records were identified through a literature search. After title and abstract screening and full-text screening, 63 studies were finally included in this meta-analysis, involving 25,577 patients (Figure 1). The included populations covered more than 30 countries around the world, of which 5 were multi-country collaborations. Forty-seven (74.6%) of the 63 included articles were prospective studies, while the remaining 16 (25.4%) were retrospective in design. The tumor spectrum included colorectal cancer (number of studies: *n* = 25, 39.7%), breast cancer (*n* = 11, 17.5%), esophagus cancer (*n* = 4, 6.3%), gastric cancer (*n* = 3, 4.8%), and others (*n* = 9, 14.3%), the remaining 11 (17.5%) studies focused on mixed solid malignancies without distinguishing specific tumor categories. The included 63 studies consisted of 92 treatment arms, and their regimens included 5-FU/capecitabine mono chemotherapy (*n* = 20, 21.7%), 5-FU/capecitabine combined chemotherapy (*n* = 33, 35.9%), 5-FU/capecitabine based chemotherapy plus targeted therapy (*n* = 25, 27.2%), 5-FU/capecitabine based chemotherapy plus radiotherapy (*n* = 6, 6.5%), and the modified fluoropyrimidine agents S1 or TAS 102 (*n* = 2, 2.2%). According to the NIH quality assessment tools, 29 studies (46%) were rated as high quality, 34 (54%) fair quality, and none was classified as poor (high risk of bias). The detailed characteristics of each included study are shown in Table 1.

**TABLE 1:** The characteristics of the included 64 studies.

No	Author	Year	Country/Region	Sample size	Study design	Age	Gender (female%)	Tumor type	Regimen	Quality	References
1	Zafar A	2021	United States	4,019	retro	58 ± 13	0.425	Mixed malignancies	5-FU or Cap based	Good	Zafar et al. (2021)
						64 ± 13	0.414				
2	Mayer IA	2021	United States	198	pros	52 (26–76)	1	Breast cancer	Cap	Good	Mayer et al. (2021)
3	Chakravarthy AB	2020	United States	355	pros	54.3 ± 11.7	0.348	Rectal cancer	mFOLFOX	Fair	Chakravarthy et al. (2020)
						53.9 ± 9.9	0.376		mFOLFOX + Bev		
4	Dyhl-Polk A (1)	2020	Denmark	108	retro	66 (35–81)	0.454	Colorectal or anal cancer	Coloretal cancer: 5-Fu or FOLFOX	Fair	Dyhl-Polk et al. (2020a)
									Metastatic: FOLFOX or FOLFIRI ± Cet or Pan		
									Anal cancer: FP + RT		
5	Delaloge S	2020	Multi-country	628	pros	18–70	1	Breast cancer	TX	Good	Delaloge et al. (2020)
6	Grierson P	2020	United States	16	pros	66 (42–73)	0.563	Pancreatic ductal adenocarcinoma	Cap + Tosedostat	Fair	Grierson et al. (2020)
7	Dyhl-Polk A (2)	2020	Denmark	2,236	retro	65 (21–85)	0.447	Colorectal cancer	5-FU based	Good	Dyhl-Polk et al. (2020b)
						70 (22–93)	0.471		Cap based		
8	Raber I	2019	United States	177	retro	54–77	0.452	Mixed malignancies	5-FU or Cap based	Fair	Raber et al. (2019)
9	Jin X	2019	China	129	retro	>18	0.217	Gastric cancer	5-FU or Cap or S-1 based	Fair	Jin et al. (2019)
10	Primrose JN	2019	United Kingdom	213	pros	62 (55–68)	0.5	Biliary tract cancer	Cap	Good	Primrose et al. (2019)
11	Abdel-Rahman O	2019	Canada	3,223	pros	60.7 (11.4)	0.403	Colorectal cancer	FOLFOX or 5-FU based + Bev and/or Pan	Good	Abdel-Rahman, (2019)
12	Hayashi Y	2019	Japan	80	pros	66.5 (62–73)	0.113	Esophageal cancer	5-FU/cisplatin + RT	Fair	Hayashi et al. (2019)
13	Peng J	2018	China	527	pros	57 (23–87)	0.339	Mixed malignancies	5-FU or Cap based	Good	Peng et al. (2018)
14	Chen EY	2018	China	47	pros	59.7 (21.4–80.1)	0.276	Colorectal cancer	FOLFIRI + Celecoxib	Good	Chen et al. (2018)
15	Kwakman JJM	2017	Netherlands	1973	pros	NA	NA	Colorectal cancer	Cap mono or based ± Bev	Good	Kwakman et al. (2017)
16	Turan T	2017	Turkey	32	pros	57	0.303	Mixed malignancies	5-FU based	Good	Turan et al. (2017)
17	Leicher LW	2017	Netherland	86	retro	69 (45–83)	0.523	Colorectal cancer	Cap	Fair	Leicher et al. (2017)
18	Zhang P	2017	China	397	pros	25–70	1	Breast cancer	Cap + Utidelone	Good	Zhang et al. (2017)
									Cap		
19	Kerr RS	2016	Multi-country	1941	pros	65 (58–71)	0.427 0.429	Colorectal cancer	Cap + Bev	Good	Kerr et al. (2016)
									Cap		
20	Winther SB	2016	Norway	71	retro	67–87	0.408	Colorectal cancer	SOX or S-1	Fair	Winther et al. (2016)
21	Polk A	2016	Denmark	452	retro	63 (28–88)	1	Breast cancer	Cap + Tra	Fair	Polk et al. (2016)
22	Mayer RJ	2015	United States	534	pros	63 (27–82)	0.389	Colorectal cancer	TAS102	Good	Mayer et al. (2015)
23	Lestuzzi C	2014	Germany	358	pros	57.5 (23–80)	NA	Mixed malignancies	5-FU or 5-FU based	Fair	Lestuzzi et al. (2014)
24	Tonyali O	2013	Turkey	37	retro	46 (30–75)	1	Breast cancer	TX + Tra	Fair	Tonyali et al. (2013)
25	Okines AFC	2013	United Kingdom	120	pros	62 (56–67)	0.321	Gastro-esophageal adenocarcinoma	ECX	Good	Okines et al. (2013)
						64 (56–69)	0.182		ECX + Bev		
26	Khan MA	2012	Pakistani	301	retro	47 (18–81)	0.249	Mixed malignancies	5-FU or 5-FU/Cap based	Fair	Khan et al. (2012)
27	Martin M	2012	Multi-country	88	Pros	53 (32–82)	0.988	Breast cancer	Cap + Bev + Tra	Fair	Martin et al. (2012)
28	Petrini L	2012	Italy	39	pros	67 (41–83)	0.154	Hepatocellular carcinoma	5-FU + Sorafenib	Good	Petrini et al. (2012)
29	Koca D	2011	Turkey	52	pros	59	0.75	Mixed malignancies	Cap or Cap based + Lap	Fair	Koca et al. (2011)
30	Jensen SA	2010	Denmark	106	pros	64 (37–81)	0.556	Colorectal cancer	FOLFOX4	Good	Jensen et al. (2010)
31	Masi G	2010	Italy	57	pros	61 (34–75)	0.4	Colorectal cancer	FOLFOXIRI + Bev	Good	Masi et al. (2010)
32	Michalaki V	2010	Greece	29	pros	52 (34–70)	1	Breast cancer	TX + Tra	Good	Michalaki et al. (2010)
33	Chua YJ	2010	Australia	105	pros	64 (54–70)	0.46	Rectal cancer	XELOX	Good	Chua et al. (2010)
34	Baur M	2010	Austria	71	pros	62 (39–84)	0.394	Rectal cancer	5-FU based	Fair	Baur et al. (2010)
35	Joensuu H	2009	Multi-country	231	pros	≤65	1	Breast cancer	5-FU based + Tra	Good	Joensuu et al. (2009)
									5-FU based		
36	Skof E	2009	Slovenia	87	pros	63 (47–75)	0.366	Colorectal cancer	XELIRI	Good	Skof et al. (2009)
						62 (34–75)			FOLFIRI		
37	Ardavanis A	2008	Greece	34	retro	69.5 (37–83)	0.47	Colorectal cancer	CapIRI + Bev	Fair	Ardavanis et al. (2008)
38	Kosmas C	2008	Greece	644	pros	66 (56–70)	NA	Mixed malignancies	5-FU based or Cap based	Good	Kosmas et al. (2008)
39	Yamamoto D	2008	Japan	59	pros	55 (42–70)	1	Breast cancer	Cap + Tra	Good	Yamamoto et al. (2008)
40	Machiels JP	2007	Belgium	40	pros	61 (34–78)	0.33	Rectal cancer	Cap + Cet + RT	Fair	Machiels et al. (2007)
41	Giantonio BJ	2007	United States	572	pros	62 (21–85)	0.395	Colorectal cancer	FOLFOX4 +Bev	Good	Giantonio et al. (2007)
						60.8 (25–84)	0.392		FOLFOX4		
42	Yilmaz U	2007	Turkey	27	pros	54 (19–70)	0.444	Gastrointestinal cancer	LV5FU2	Fair	Yilmaz et al. (2007)
43	Emmanouilides C	2007	Greece	53	pros	65 (18–78)	0.434	Colorectal cancer	FOLFOX + Bev	Fair	Emmanouilides et al. (2007)
44	Geyer CE	2006	United States	324	pros	54 (26–80)	1	Breast cancer	Cap + Lap	Good	Geyer et al. (2006)
						51 (28–83)			Cape		
45	Mambrini A	2006	Italy	211	pros	61 (21–79)	NA	Pancreatic cancer	FEC	Good	Mambrini et al. (2006)
46	Koopman M	2006	Netherland	393	pros	64 (27–84)	0.373	Colorectal cancer	Cap	Good	Koopman et al. (2006)
						63 (35–79)	0.396		CapIRI		
47	Jensen SA	2006	Denmark	668	retro	NA	NA	Colorectal or gastric cancers	Cap	Fair	Jensen and Sorensen, (2006)
									Cap/Capatin/Docetaxel		
									5-FU		
									LV5FU2		
									FOLFOX-4		
48	Yerushalmi R	2006	Israel	89	retro	66 (25–82)	0.418	Rectal cancer	Cap + RT	Fair	Yerushalmi et al. (2006)
						62 (23–81)	0.5		5-FU + RT		
49	Giordano KF	2006	United States	44	pros	57 (32–77)	0.114	Gastric or gastro-esophageal junction adenocarcinoma	TX	Fair	Giordano et al. (2006)
50	Jatoi A	2006	United States	46	pros	61 (32–80)	0.116	Esophageal or gastro-esophageal junction adenocarcinoma	XELOX	Fair	Jatoi et al. (2006)
51	Baghi M	2006	Germany	24	pros	60 (23–79)	0.042	Head and neck squamous cell carcinoma	TPF	Fair	Baghi et al. (2006)
562	Meydan N	2005	Turkey	231	retro	59 (23–87)	0.402	Mixed malignancies	LV5FU2	Fair	Meydan, (2005)
53	Lordick F	2005	Germany	48	pros	62 (41–75)	0.187	Gastric cancer	FUFOX	Fair	Lordick et al. (2005)
54	Ng M	2005	United Kingdom	153	pros	33–81	0.412	Colorectal cancer	CapeOx	Good	Ng et al. (2005)
55	Feliu J	2005	Spain	51	pros	76 (71–89)	0.392	Colorectal cancer	Cap	Fair	Feliu et al. (2005)
56	Wacker A	2003	Germany	102	pros	61.7 (39–78)	0.311	Mixed malignancies	5-FU or 5-FU based	Fair	Wacker et al. (2003)
57	Vaishampayan UN	2002	United States	32	retro	67.5 (45–84)	0.375	Gastrointestinal cancer	Cap + RT	Fair	Vaishampayan et al. (2002)
58	Tsavaris N	2002	Greece	427	retro	NA	NA	Mixed malignancies	5-Fu based	Fair	Tsavaris et al. (2002)
59	Van Cutsem E	2002	Switzerland	1,425	pros	NA	NA	Colorectal cancer	LV5FU2	Fair	Van Cutsem et al. (2002)
						NA		Colorectal cancer	Cap		
						NA		Breast cancer	Cap		
60	Hartung G	2001	Germany	51	pros	60 (24–77)	0.25	Colorectal cancer	LV5FU2	Fair	Hartung et al. (2001)
61	Dencausse Y	2001	Germany	21	pros	30–80	0.333	Rectal cancer	LV5FU2+RT	Fair	Dencausse et al. (2001)
62	Peiffert D	2001	France	80	pros	≤75	0.837	Anal cancer	FP + RT	Fair	Peiffert et al. (2001)
63	Hoff PM	2001	Multi-country	605	pros	64 (23–86)	0.40	Colorectal cancer	Cap	Good	Hoff, (2001)
						63 (24–87)	0.35		LV5FU2		

Notes: a, Mixed malignancies: including two or more tumor types, such as breast cancer, colorectal cancer, gastric cancer, head and neck cancer, and so on; NA: not available; RT: radiotherapy; Cap: Capecitabine; Bev: Bevacizumab; Cet: Cetuximab; Pan: Panitumumab; Tra, Trastuzumab; Lap, Lapatinib.

### The Incidence of 5-Fluorouracil Associated Coronary Artery Disorders

**FIGURE 2:**
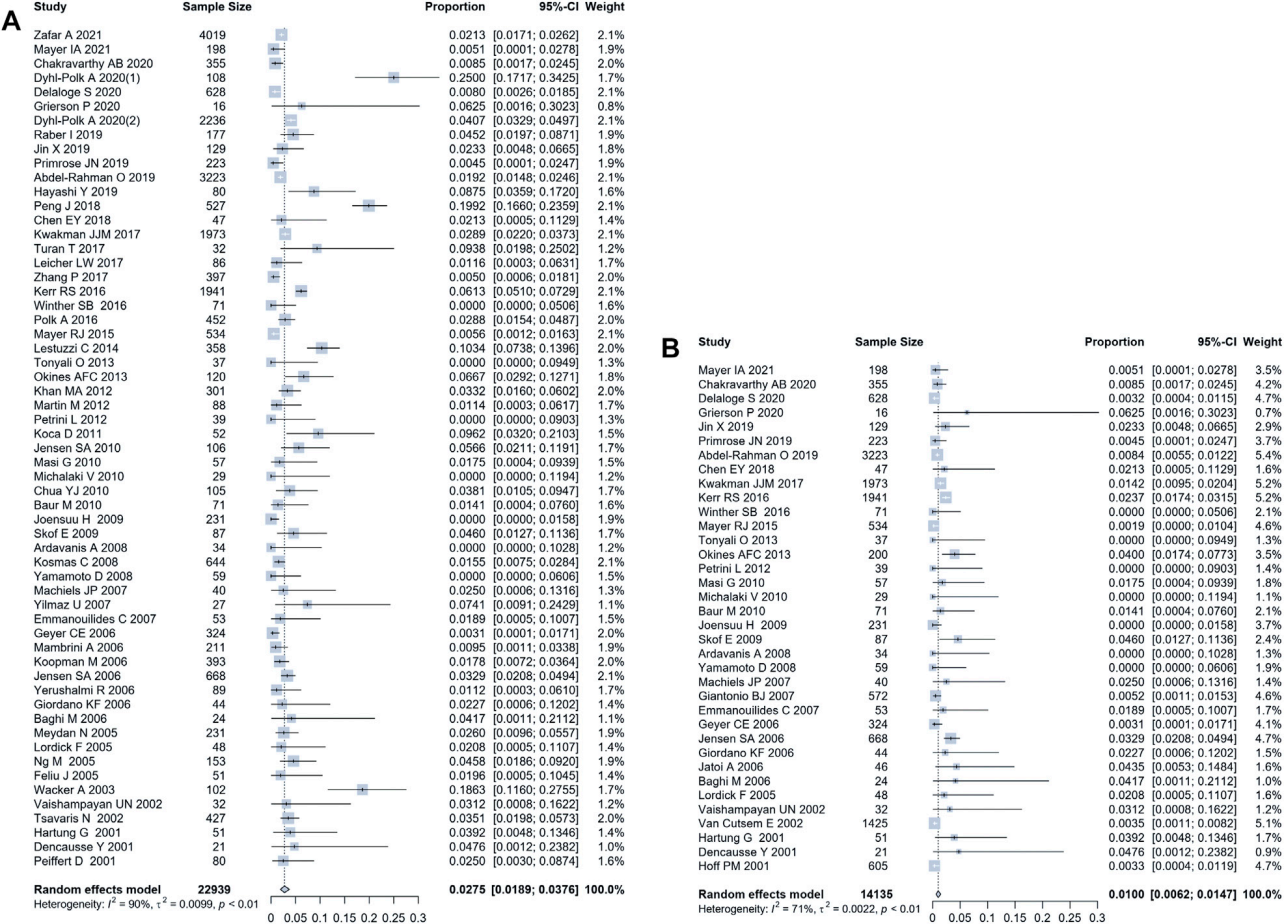
Forest plot of the incidence of fluoropyrimidine-related coronary disorders. **(A)** the pooled incidence of all-grade adverse coronary events, by a random-effect model analysis, was 2.75% (95% CI 1.89%–3.76%); **(B)** the pooled incidence of grade 3 or higher adverse coronary events, by a random-effect model analysis, was and 1.00% (95% CI 0.62%–1.47%).

Using a random-effect model, the pooled incidence of all-grade fluoropyrimidine-related coronary disorders among 22,939 cases from 59 studies was 2.75% (95% CI 1.89%–3.76%) (Figure 2A). Thirty-three studies reported the incidence of grade 3 or higher fluoropyrimidine-related coronary disorders, involving a total of 14,135 cases, The pooled incidence of grade 3 or higher coronary disorders by meta-analysis, was 1.00% (95% CI 0.62%–1.47%) (Figure 2B).

### Specific Reported Events of Coronary Disorders

**FIGURE 3:**
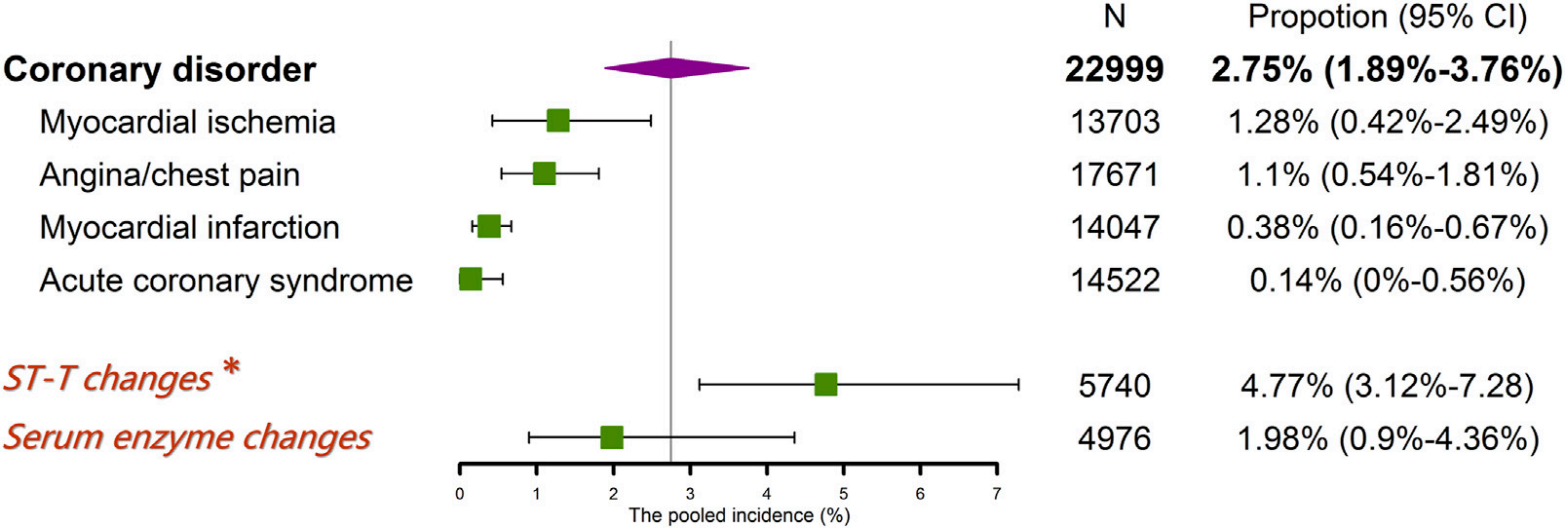
The pooled incidence of specific reported events of coronary disorders. * a pooled incidence of 4.77% (95% CI 3.12%–7.28%), containing ST-T changes on ECG with or without symptomatic coronary toxicities.

Coronary disorders were frequently reported as angina/chest pain, myocardial infarction, myocardial ischemia, and acute coronary syndrome in our included literature. As shown in Figure 3, myocardial ischemia and angina/chest pain were the two most common adverse events, which have a pooled incidence of 1.28% (95% CI 0.42%–2.49%) and 1.1% (95% CI 0.54%–1.81%), respectively. Myocardial infarction and the acute coronary syndrome were less reported, with a pooled incidence of 0.38% (95% CI 0.16%–0.67%) and 0.14% (0–0.56%), respectively. Fourteen studies reported the typical ST-T changes on ECG with or without symptomatic coronary toxicities. A random-effect meta-analysis gave a pooled incidence of ST-T changes of 4.77% (95% CI 3.12%–7.28%), significantly higher than the incidence of adverse coronary events (2.75%). The changes of cardiac-specific serum enzymes were reported in 10 studies, including troponin, CK-MB, myoglobin, BNP, and copeptin, and the pooled overall incidence was 1.98% (95% CI 0.9%–4.36%).

### Subgroup Analyses

Subgroup analyses were conducted to compare the incidence of all-grade and grade 3 or higher coronary disorders among different study-level moderators, and further identify the factors influencing the occurrence of adverse coronary events. The pooled incidence and 95% CI of coronary events in each subgroup were shown in Table 2, as well as the results of statistical comparisons between subgroups. A significant difference was identified among different publication periods (*p* = 0.02) for the incidence of all-grade coronary events, but not statistically significant for grade 3 or higher events (*p* = 0.65). We did not observe an obvious difference between prospective and retrospective study designs (all-grade: *p* = 0.58, grade 3 or higher: *p* = 0.21), nor between phase Ⅱ and phase Ⅲ clinical trials (all-grade: *p* = 0.24, grade 3 or higher: *p* = 0.18). There was also no significant difference between studies with good-quality and fair-quality (*p* = 0.43) for all-grade events, however, the good-quality studies had lower pooled incidence than fair-quality studies for the assessment of grade 3 or higher coronary events (*p* < 0.01). Notably, the female-only population (with breast cancer) reported lower pooled incidence than general populations, both in the assessment of all-grade (*p* < 0.01) and grade 3 or higher (*p* < 0.01) coronary disorders.

**TABLE 2:** The pooled incidence of coronary disorder in each subgroup and the comparison results.

Subgroup	All-grade adverse coronary events			Grade 3 or higher adverse coronary events		
	Sample size (*N*)	Incidence (95%CI)	Comparison results	Sample size (*N*)	Incidence (95%CI)	Comparison results
Publication period						
2001–2005	1,196	4.27% (2.16%–7.06%)	χ^2^ = 10.15, *p* = 0.02*	1,329	0.92% (0.00%–3.26%)	χ^2^ = 1.64, *p* = 0.65
2006–2010	3,190	1.28% (0.65%–2.13%)		1767	1.12% (0.25%–2.40%)	
2011–2015	1,635	3.05% (0.93%–6.31%)		810	0.58% (0.00%–3.11%)	
2016–2022	16,978	3.37% (1.66%–5.65%)		8,804	0.72% (0.29%–1.28%)	
Study design						
Prospective study	13,950	3.02% (1.88%–4.42%)	χ^2^ = 0.31, *p* = 0.58	11,739	0.67% (0.26%–1.20%)	χ^2^ = 1.55, *p* = 0.21
Retrospective study	9,049	2.62% (1.98%–3.34%)		971	1.42% (0.30%–3.08%)	
Phase for clinical trials						
Ⅱ	938	1.93% (1.14%–2.92%)	χ^2^ = 1.41, *p* = 0.24	711	0.15% (0.38%–2.62%)	χ^2^ = 1.76, *p* = 0.18
Ⅲ	7,617	1.18% (0.49%–2.16%)		8,576	0.69% (0.29%–1.09%)	
Study quality						
Good	18,385	2.12% (1.08%–3.48%)	χ^2^ = 1.67, *p* = 0.43	10,970	0.58% (0.20%–1.10%)	χ^2^ = 9.32, *p*＜0.01*
Fair	4,162	3.35% (2.03%–4.98%)		1740	1.51% (0.70%–2.54%)	
Age						
No limitation	22,797	2.75% (1.87%–3.79%)	χ^2^ = 0.04, *p* = 0.84	12,639	0.78% (0.35%–1.33%)	χ^2^ = 1.07, *p* = 0.30
Old	202	2.17% (0.00%–10.00%)		71	0.00% (0.00%–5.06%)	
Gender						
All	20,556	3.48% (2.44%–4.70%)	χ^2^ = 18.59, *p* < 0.01*	11,204	1.09% (0.53%–1.78%)	χ^2^ = 15.75, *p* < 0.01*
Female-only	2,355	0.61% (0.15%–1.37%)		1,418	0.09% (0.00%–0.43%)	
Tumor type						
Esophagus cancer	244	6.32% (3.62%–9.71%)	χ^2^ = 47.59, *p*＜0.01*	290	3.51% (1.51%–6.14%)	χ^2^ = 34.41, *p*＜0.01*
Colorectal cancer	12,553	2.69% (1.57%–4.09%)		10,403	0.94% (0.39%–1.67%)	
Gastric cancer	177	2.26% (0.59%–4.96%)		177	2.13% (0.31%–5.05%)	
Pancreatic cancer	227	1.64% (0.00%–6.13%)		16	6.25% (0.16%–30.23%)	
Breast cancer	2,443	0.50% (0.11%–1.16%)		1,506	0.01% (0.00%–0.27%)	
Biliary tract cancer	223	0.45% (0.01%–2.47%)		223	0.45% (0.01%–2.47%)	
Others^a^						
Treatment type						
Adjuvant	3,703	1.36% (0.16%–3.36%)	χ^2^ = 2.01, *p* = 0.37	3,366	0.94% (0.32%–1.88%)	χ^2^ = 3.84, *p* = 0.15
Neoadjuvant	549	2.86% (1.50%–4.56%)		380	2.65% (1.16%–4.71%)	
For advanced/metastasis/relapse disease	925	1.70% (0.72%–2.97%)		933	1.10% (0.27%–2.48%)	
Regimen						
5-FU monotherapy	484	3.31% (1.46%–5.87%)	χ^2^ = 28.65, *p* < 0.01*	1,380	0.92% (0.00%–3.04%)	χ^2^ = 15.79, *p* = 0.07
Capecitabine monotherapy	2,627	1.21% (0.34%–2.59%)		3,059	0.75% (0.03%–1.36%)	
5-FU combined chemotherapy	2,993	4.31% (2.05%–7.35%)		706	1.2% (0.00%–4.31%)	
Capecitabine combined chemotherapy	3,956	2.69% (1.09%–4.98%)		1711	0.69% (0.00%–2.14%)	
5-FU based/targeted therapy	336	1.46% (0.46%–3.02%)		623	0.83% (0.14%–1.87%)	
Capecitabine based/targeted therapy	3,177	2.85% (1.75%–4.20%)		2,483	1.22% (0.46%–2.24%)	
5-FU based/radio	181	5.10% (1.58%–10.48%)		21	4.76% (0.12%–23.82%)	
Capecitabine based/radio	75	2.65% (0.25%–7.47%)		32	3.12% (0.08%–16.22%)	
S-1	71	0.00% (0.00%–5.06%)		71	0.00% (0.00%–5.06%)	
TAS 102	534	0.56% (0.12%–1.63%)		534	0.19% (0.00%–1.04%)	

Notes: *p < 0.05; a, “others” including liver cancer, gastrointestinal cancer, and head and neck cancer.

The pooled incidence of coronary disorders for all-grade or grade 3 or higher varied between tumor types (all-grade: *p* < 0.01, grade 3 or higher: *p* < 0.01). Fluoropyrimidine-related coronary disorders were most frequently in the treatment of esophageal cancer, with the all-grade incidence of 6.32% (95% CI 3.62%–9.71%). Fluoropyrimidines in the treatment of breast cancer, however, occupied the relatively lower coronary complications (all-grade: 0.50%, 95% CI 0.11%–1.16%) than colorectal cancer (all-grade: 2.69%, 95% CI 1.57%–4.09%) and esophagus cancer.

The effect of treatment parameters on the incidence of coronary events was also analyzed. As a result, the administrations of fluoropyrimidine as neoadjuvant chemotherapy, adjuvant chemotherapy, or palliative treatment for advanced/metastasis/relapse disease did not significantly affect the occurrence of coronary events (all-grade: *p* = 0.37; grade 3 or higher: *p* = 0.15). However, the treatment regimen is closely related to the occurrence of coronary disorders (all-grade: *p* < 0.01; grade 3 or higher: *p* = 0.07). Coronary disorder induced by 5-FU is more frequent than that induced by capecitabine, both for all-grade (3.31% vs. 1.21%) and grade 3 or higher (0.92% vs. 0.75%). The 5-FU or capecitabine combined chemotherapy had a higher incidence of coronary events than 5-FU or capecitabine monotherapy (5-FU: 4.31% vs. 3.31%; capecitabine: 2.69% vs. 1.21%). The addition of targeted therapy drugs (e.g., bevacizumab, cetuximab, and trastuzumab) to capecitabine increased the risk of coronary disorder (all-grade; 2.85% vs. 1.21%; grade 3 or higher: 1.22% vs. 0.75%). Similarly, the addition of radiotherapy resulted in a significant increase in coronary toxicity, both for 5-FU (all-grade: 5.1% vs. 3.3%, grade 3 or higher: 4.76% vs. 0.92%) and capecitabine (all-grade: 2.65% vs. 1.21%, grade 3 or higher: 3.12% vs. 0.75%). Novel fluoropyrimidines, S-1 and Tas 102, demonstrated lower coronary toxicity (S-1: 0; Tas102: 0.56%), however, such data were derived from a limited number of studies.

### Sensitive Analyses and Publication Bias

**FIGURE 4:**
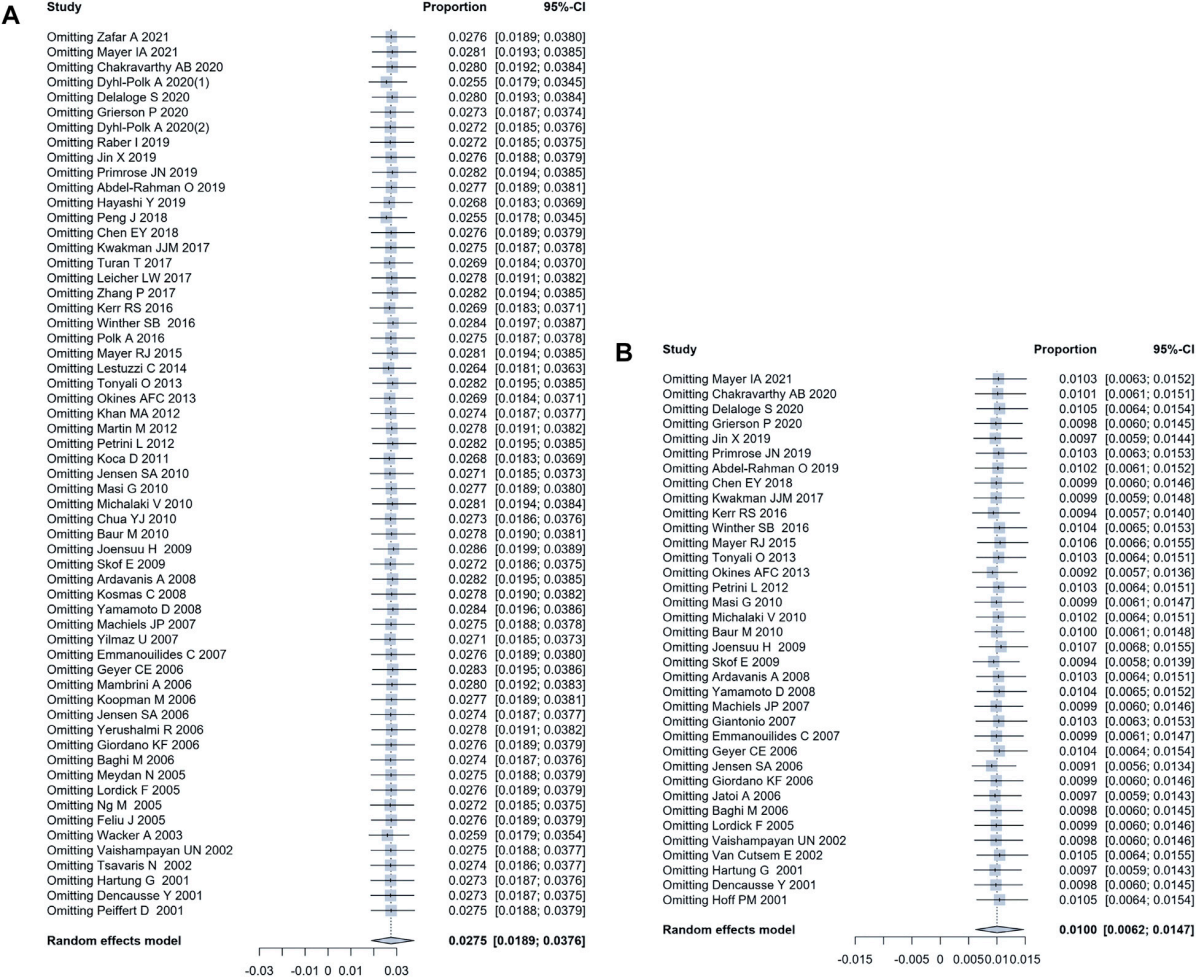
The results of sensitive analysis. **(A)** the sensitive analysis of the incidence of all-grade coronary disorders indicated a variation between 2.64% and 2.86%; **(B)** the sensitive analysis of the incidence of grade 3 or higher coronary disorders indicated a variation between 0.92% and 1.07%.

**FIGURE 5:**
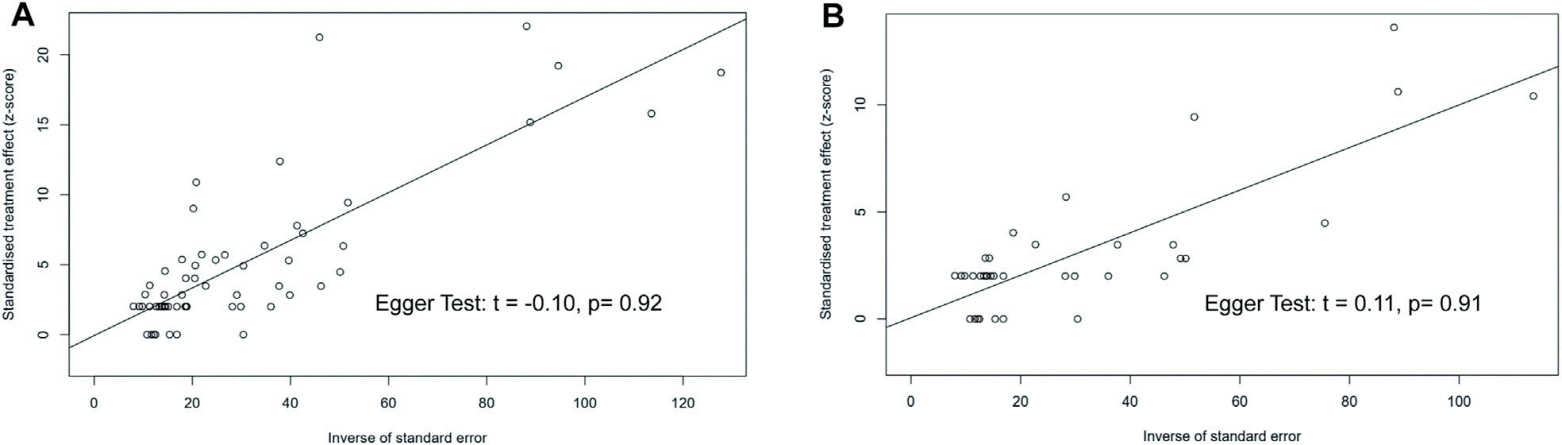
Funnel plot and Egger test evaluating the publication bias of studies. The *p*-values of Egger test for all-grade and grade 3 or higher coronary disorder were 0.92 and 0.91, respectively, suggesting no significant publication bias.

Sensitivity analyses were performed for the main outcome measures, all-grade and grade 3 or higher incidence of coronary disorders. In the all-grade and grade 3 or higher analyses, the variation of the pooled results after removing studies one by one was 2.64%–2.86% and 0.92%–1.07%, respectively (Figure 4), indicating that the conclusions of this meta-analysis were stable and reliable. The funnel plots and Egger tests did not show existing significant publication bias in the evaluation of all-grade and grade 3 or higher coronary disorder in this meta-analysis (Figure 5).

## Discussion

Fluoropyrimidine, as a well-known class of pyrimidine antimetabolites, has been used in cancer treatment for more than half a century. Although numerous therapeutic strategies have been introduced in recent years, such as targeted therapy (Bedard et al., 2020), antiangiogenic therapy, and immunotherapy (Hegde and Chen, 2020), fluoropyrimidines are still one of the most effective and frequently used agents in the treatment of colorectal cancer, breast cancer, gastric cancer, and head and neck cancers, whether for neoadjuvant, adjuvant, advanced or maintenance therapy. Cardiotoxicity, especially coronary disorders caused by 5-FU and capecitabine remains a critical issue in cancer therapy that threatens patient survival and leads to the discontinuation of the medication. Unfortunately, there is no solid evidence worldwide about the incidence of fluoropyrimidine-related coronary disorders and the risk factors affecting its occurrence (Deac et al., 2020; Li et al., 2021). In this study, we systematically evaluated the incidence and profile of coronary disorder associated with fluoropyrimidines administration. To our best knowledge, this is the first comprehensive systematic review and meta-analysis on this topic.

The mechanism of fluoropyrimidine-induced cardiotoxicity has not yet been fully elucidated. Although several theories have been proposed, including vasoconstriction, endothelial injury, direct myocardial toxicity, and so on, the most predominant and important clinicopathological change was the disorder of coronary artery (Depetris et al., 2018; Mohammed et al., 2018; Chong and Ghosh, 2019). The coronary disorders defined in this study mainly refers to reversible cardiac ischemia caused by coronary vasospasm, and coronary atherosclerosis due to fluorouracil-induced coagulation problems was also included. There are several reported presentations of fluoropyrimidine-related coronary disorders, including atypical chest pain to typical angina, ACS, myocardial ischemia, and myocardial infarction. According to our results, myocardial ischemia (1.28%) and angina/chest pain (1.1%) are the most frequently reported. In fact, ischemia and angina/chest pain are not two independent adverse events. Chest pain with or without typical angina is often the primary clinical manifestation of acute cardiac ischemia or ACS, both of which are outcomes of coronary disorders. Thus, in this analysis, we focused on the overall coronary disorders consisting of angina/chest pain, myocardial ischemia and infarction, and ACS, rather than one of them.

Our results generated reliable data on the overall incidence of fluoropyrimidine-related coronary disorder of 2.7%, which revised the previous over-or under-estimation of 0–35%. The incidence of grade 3 or higher fluoropyrimidine reached 1%, accounting for 37% of the overall incidence, indicating that coronary disorder is one of the high-risk complications, which deserves special attention. The pooled results in our study were close to the data reported by Zafar et al. (2021), in which coronary disorders occurred in 2.16% of 4,019 patients treated with 5-FU. It should be noted that 14 of the 63 included studies observed ECG changes during fluoropyrimidine administration, with a pooled incidence of ST-T changes of 4.77%, remarkably exceeding the incidence of adverse coronary events (2.16%). Such inconsistency may be derived from the presence of asymptomatic ischemic ECG changes in some populations (Lounsbury et al., 2017). Therefore, continuous ECG monitoring should be recommended during fluoropyrimidine use, as early ST-T changes often indicate an impending adverse coronary event.

The results of our subgroup analysis showed a lower incidence of the coronary disorder in the female-only population, a phenomenon that has also been observed in other studies (Peng et al., 2018). Delaloge et al. (2020) reported 5 (0.8%) of 628 breast cancer patients treated with capecitabine developed coronary disorders in a phase Ⅲ clinical trial. A similar low incidence (0.5%, 2/397) was also reported by Zhang et al. (2017) in 2017. Such gender differences may be associated with the protective effect of female hormones on the heart (Kurokawa et al., 2009; Gowd and Thompson, 2012; Costa et al., 2021). However, in this pooled analysis, the female-only population were breast cancer patients with capecitabine administration. We believed that the characteristics in tumor type and medication should be mainly accounted for the lower coronary toxicity in the female-only population. In addition, a significant difference on the incidence of all-grade adverse coronary events was also observed among different publication periods. This discrepancy could be partly related to the way of drug administration, increased concomitant targeted therapy, and increased attention to cardiotoxicity.

We had observed a significant difference in fluoropyrimidine-related coronary disorders among different tumor types. However, these differences, to a great extent, should be attributed to the variability in treatment regimens among tumors. Capecitabine is an oral prodrug of 5-FU designed to be converted selectively in tumors. It is rapidly absorbed from the gut as an unchanged drug and then converted to the active form of 5-FU by carboxylesterase and thymidine phosphorylase (O’Connell et al., 2014). Therefore, the effect of capecitabine on the coronary is indirect, and our results seem to show that the incidence of capecitabine-caused coronary disorders is significantly lower than that of intravenous 5-FU. However, due to the lack of evidence of direct comparison between 5-FU and capecitabine, such a conclusion needs further confirmation. The coronary toxicity was distinctly varied from formulations or administration protocols of 5-FU or capecitabine. Combination therapy significantly increases coronary toxicity, whether combined with other chemotherapeutics or targeted therapy. The increased incidence of the coronary disorder in combination therapy may result from additive and synergistic toxic effects of different agents on the heart. As we know, anti-angiogenic targeted drugs (e.g., bevacizumab) also had adverse effects on the cardiovascular system (Economopoulou et al., 2015). Therefore, when combination regimens containing these agents were considered, more attention should be paid to the occurrence of coronary adverse events. On the other hand, radiotherapy covering or adjacent to the heart also significantly increases coronary toxicity of fluoropyrimidines. As in our meta-analysis, patients with esophageal cancer who received 5-FU combined with radiotherapy had the highest incidence of coronary disorder at 6.32%. Some studies further showed that radiotherapy increases not only short-term cardiotoxicity, but also long-term cardiotoxicity, such as pericarditis and pericardial effusion (Saunders and Anwar, 2019). Other fluoropyrimidine drugs, such as S-1 and TAS102, have shown a lower incidence of coronary disorders in our study and may be a safer option for patients. However, due to the limited number of cases included in the TAS102 and S1 analyses, more evidence is needed.

Admittedly, there were some limitations in this meta-analysis. First, heterogeneity was observed among the included studies. Although we have performed subgroup analyses and adopted a random-effect model to minimize the effects of the heterogeneity, its influence on the stability of the results cannot be eliminated. Second, it is difficult to clearly define and distinguish “coronary disorder,” although in this study we included various manifestations such as angina, chest pain, myocardial infarction, myocardial ischemia, and ACS. Not all included studies have undertaken a comprehensive and targeted examination to identify these conditions, so the result may be an inevitable underestimation of the incidence. Furthermore, it is difficult to determine whether the referred coronary disorder was related to fluoropyrimidine-containing treatment. Although we only included studies that clearly indicated such a correlation, there is still a possibility that patients with spontaneous coronary disorder could be counted in the original study. Finally, several previous studies have reported the effects of age, race, smoking, history of heart disease, and other factors on fluoropyrimidine-related coronary toxicity. However, limited by the characteristics of the included studies in this meta-analysis, we did not have enough data to further analyze all possible moderators. Owing to the above limitations, the findings of this meta-analysis should be interpreted with carefully, and subsequent large-sample clinical studies are necessary.

## Conclusion

In conclusion, this meta-analysis, which used a single-rate pooled analysis model, has defined the incidence of coronary disorders induced by fluoropyrimidine-based treatment, and depicted its epidemiological profiles. The occurrence of fluoropyrimidine-related coronary disorders is not a rare condition during fluoropyrimidine administration, which needs to be highly concerned. It varies among tumor types, and different treatment regimens may be associated with different incidence of adverse coronary events. This comprehensive overview of fluoropyrimidine-related coronary disorders can provide a reference for clinical practice in cancer management.

## Data Availability

The original contributions presented in the study are included in the article/Supplementary Material, further inquiries can be directed to the corresponding author.

## References

[B1] Abdel-Rahman O. (2019). 5-Fluorouracil-related Cardiotoxicity; Findings from Five Randomized Studies of 5-Fluorouracil-Based Regimens in Metastatic Colorectal Cancer. Clin. Colorectal Cancer 18 (1), 58–63. 10.1016/j.clcc.2018.10.006 30470591

[B2] Ardavanis A. Kountourakis P. Mantzaris I. Malliou S. Doufexis D. Sykoutri D. (2008). Bevacizumab Added to the Irinotecan and Capecitabine Combination for Advanced Colorectal Cancer: A Well-Tolerated, Active and Convenient Regimen. Anticancer Res. 28 (5B), 3087–3092. 10.1200/jco.2008.26.15_suppl.15085 19031962

[B3] Baghi M. Hambek M. Wagenblast J. May A. GstoettnerGstoettner W. Knecht R. (2006). A Phase II Trial of Docetaxel, Cisplatin and 5-fluorouracil in Patients with Recurrent Squamous Cell Carcinoma of the Head and Neck (SCCHN). Anticancer Res. 26 (1B), 585–590. 10.1016/j.drugalcdep.2015.07.446 16739325

[B4] Baldeo C. Baldeo C. Mody K. Seegobin K. Rollini F. (2018). A Case of 5-Fluorouracil-Induced Coronary Artery Vasovasospasm in Rectal Adenocarcinoma. J. Am. Coll. Cardiol. 71S (11), 2324. 10.1016/S0735-1097(18)32865-1

[B5] Baur M. Horvath M. Stättner S. Schratter-Sehn A. Horvath B. Sellner F. (2010). Chemoradiotherapy with 5-fluorouracil/leucovorin, Surgery and Adjuvant Chemotherapy for Locally Advanced Rectal Cancer. Oncol. Lett. 1 (1), 189–194. 10.3892/ol_00000035 22966281 PMC3436440

[B6] Bedard P. L. Hyman D. M. Davids M. S. Siu L. L. (2020). Small Molecules, Big Impact: 20 Years of Targeted Therapy in Oncology. Lancet 395 (10229), 1078–1088. 10.1016/S0140-6736(20)30164-1 32222192

[B7] Ben-Yakov M. Mattu A. Brady W. J. Dubbs S. B. (2017). Prinzmetal Angina (Coronary Vasospasm) Associated with 5-fluorouracil Chemotherapy. Am. J. Emerg. Med. 35 (e37), 1038–e5. 10.1016/j.ajem.2017.02.046 28283339

[B8] Chakravarthy A. B. Zhao F. Meropol N. J. Flynn P. J. Wagner L. I. Sloan J. (2020). Intergroup Randomized Phase III Study of Postoperative Oxaliplatin, 5-Fluorouracil, and Leucovorin versus Oxaliplatin, 5-Fluorouracil, Leucovorin, and Bevacizumab for Patients with Stage II or III Rectal Cancer Receiving Preoperative Chemoradiation: A Trial of the ECOG-ACRIN Research Group (E5204). Oncologist 25 (5), e798. 10.1634/theoncologist.2019-0437 31852811 PMC7216434

[B9] Chen E. Y. Blanke C. D. Haller D. G. Benson A. B. Dragovich T. Lenz H. J. (2018). A Phase II Study of Celecoxib with Irinotecan, 5-Fluorouracil, and Leucovorin in Patients with Previously Untreated Advanced or Metastatic Colorectal Cancer. Am. J. Clin. Oncol. 41 (12), 1193–1198. 10.1097/COC.0000000000000465 29782360 PMC6240505

[B10] Chong J. H. Ghosh A. K. (2019). Coronary Artery Vasospasm Induced by 5-fluorouracil: Proposed Mechanisms, Existing Management Options and Future Directions. Interv. Cardiol. 14 (2), 89–94. 10.15420/icr.2019.12 31178935 PMC6545978

[B11] Chua Y. J. Barbachano Y. Cunningham D. Oates J. R. Brown G. Wotherspoon A. (2010). Neoadjuvant Capecitabine and Oxaliplatin before Chemoradiotherapy and Total Mesorectal Excision in MRI-Defined Poor-Risk Rectal Cancer: a Phase 2 Trial. Lancet Oncol. 11 (3), 241–248. 10.1016/S1470-2045(09)70381-X 20106720

[B12] Costa S. Saguner A. M. Gasperetti A. Akdis D. Brunckhorst C. Duru F. (2021). The Link between Sex Hormones and Susceptibility to Cardiac Arrhythmias: From Molecular Basis to Clinical Implications. Front. Cardiovasc. Med. 8, 644279. 10.3389/fcvm.2021.644279 33681311 PMC7925388

[B13] Curigliano G. Cardinale D. Dent S. Criscitiello C. Aseyev O. Lenihan D. (2016). Cardiotoxicity of Anticancer Treatments: Epidemiology, Detection, and Management. CA Cancer J. Clin. 66 (4), 309–325. 10.3322/caac.21341 26919165

[B14] Das S. K. Das A. K. William M. (2019). 5-Fluorouracil-induced Acute Coronary Syndrome. Med. J. Aust. 211 (6), 255–e1. 10.5694/mja2.50317 31441056

[B15] Deac A. L. Burz C. C. Bocsan I. C. Buzoianu A. D. (2020). Fluoropyrimidine-induced Cardiotoxicity. World J. Clin. Oncol. 11 (12), 1008–1017. 10.5306/wjco.v11.i12.1008 33437663 PMC7769712

[B16] Delaloge S. Piccart M. Rutgers E. Litière S. van 't Veer L. J. van den Berkmortel F. (2020). Standard Anthracycline Based versus Docetaxel-Capecitabine in Early High Clinical And/or Genomic Risk Breast Cancer in the EORTC 10041/BIG 3-04 MINDACT Phase III Trial. J. Clin. Oncol. 38 (11), 1186–1197. 10.1200/JCO.19.01371 32083990 PMC7840116

[B17] Dencausse Y. Sturm J. Hartung G. Dietzler P. Edler L. Bambach M. (2001). Adjuvant Radio-Chemotherapy in Stage II-III Rectal Cancer with 24-hour Infusion of High-Dose 5-fluorouracil and Folinic Acid: Evaluation of Feasibility. Onkologie 24 (5), 476–480. 10.1159/000055129 11694775

[B18] Depetris I. Marino D. Bonzano A. Cagnazzo C. Filippi R. Aglietta M. (2018). Fluoropyrimidine-induced Cardiotoxicity. Crit. Rev. Oncol. Hematol. 124, 1–10. 10.1016/j.critrevonc.2018.02.002 29548480

[B19] Dyhl-Polk A. Schou M. Vistisen K. K. Sillesen A. S. Serup-Hansen E. Faber J. (2020a). Myocardial Ischemia Induced by 5-Fluorouracil: A Prospective Electrocardiographic and Cardiac Biomarker Study. Oncologist 26 (3), E403–E413. 10.1002/onco.13536 32959474 PMC7930422

[B20] Dyhl-Polk A. Vaage-Nilsen M. Schou M. Vistisen K. K. Lund C. M. Kümler T. (2020b). Incidence and Risk Markers of 5-fluorouracil and Capecitabine Cardiotoxicity in Patients with Colorectal Cancer. Acta Oncol. 59 (4), 475–483. 10.1080/0284186X.2019.1711164 31931649

[B21] Economopoulou P. Kotsakis A. Kapiris I. Kentepozidis N. (2015). Cancer Therapy and Cardiovascular Risk: Focus on Bevacizumab. Cancer Manag. Res. 7, 133–143. 10.2147/CMAR.S77400 26082660 PMC4461138

[B22] Emmanouilides C. Sfakiotaki G. Androulakis N. Kalbakis K. Christophylakis C. Kalykaki A. (2007). Front-line Bevacizumab in Combination with Oxaliplatin, Leucovorin and 5-Fluorouracil (FOLFOX) in Patients with Metastatic Colorectal Cancer: a Multicenter Phase II Study. BMC Cancer 7 (91), 91. 10.1186/1471-2407-7-91 17537235 PMC1894803

[B23] Feliu J. Escudero P. Llosa F. Bolaños M. Vicent J. M. Yubero A. (2005). Capecitabine as First-Line Treatment for Patients Older Than 70 Years with Metastatic Colorectal Cancer: An Oncopaz Cooperative Group Study. J. Clin. Oncol. 23 (13), 3104–3111. 10.1200/JCO.2005.06.035 15860870

[B24] Gao L. Tatsch T. Sides M. Willis M. Berbarie R. (2019). 5-fluorouracil Induced Coronary Vasospasm and Non-ischemic Cardiomyopathy Presenting in the Same Patient. J. Am. Coll. Cardiol. 73 (9), 2477. 10.1016/S0735-1097(19)33083-9 31097169

[B25] Geyer C. E. Forster J. Lindquist D. Chan S. Romieu C. G. Pienkowski T. (2006). Lapatinib Plus Capecitabine for HER2-Positive Advanced Breast Cancer. N. Engl. J. Med. 355 (26), 2733–2743. 10.1056/NEJMoa064320 17192538

[B26] Giantonio B. J. Catalano P. J. Meropol N. J. O'Dwyer P. J. Mitchell E. P. Alberts S. R. (2007). Bevacizumab in Combination with Oxaliplatin, Fluorouracil, and Leucovorin (FOLFOX4) for Previously Treated Metastatic Colorectal Cancer: Results from the Eastern Cooperative Oncology Group Study E3200. J. Clin. Oncol. 25 (12), 1539–1544. 10.1200/JCO.2006.09.6305 17442997

[B27] Giordano K. F. Jatoi A. Stella P. J. Foster N. Tschetter L. K. Alberts S. R. (2006). Docetaxel and Capecitabine in Patients with Metastatic Adenocarcinoma of the Stomach and Gastroesophageal junction: a Phase II Study from the North Central Cancer Treatment Group. Ann. Oncol. 17 (4), 652–656. 10.1093/annonc/mdl005 16497828

[B28] Gowd B. M. Thompson P. D. (2012). Effect of Female Sex on Cardiac Arrhythmias. Cardiol. Rev. 20 (6), 297–303. 10.1097/CRD.0b013e318259294b 22531673

[B29] Grierson P. Teague A. Suresh R. Lim K. H. Amin M. Pedersen K. (2020). Phase Ib/II Study Combining Tosedostat with Capecitabine in Patients with Advanced Pancreatic Adenocarcinoma. J. Gastrointest. Oncol. 11 (1), 61–67. 10.21037/jgo.2019.11.06 32175106 PMC7052765

[B30] Hartung G. Hofheinz R. D. Wein A. Riedel C. Rost A. Fritze D. (2001). Phase II Study of a Weekly 24-hour Infusion with 5-fluorouracil and Simultaneous Sodium-Folinic Acid in the First-Line Treatment of Metastatic Colorectal Cancer. ONKOLOGIE 24 (5), 457–462. 10.1159/000055126 11694772

[B31] Hayashi Y. Iijima H. Isohashi F. Tsujii Y. Fujinaga T. Nagai K. (2019). The Heart's Exposure to Radiation Increases the Risk of Cardiac Toxicity after Chemoradiotherapy for Superficial Esophageal Cancer: a Retrospective Cohort Study. BMC Cancer 19 (1), 195. 10.1186/s12885-019-5421-y 30832605 PMC6399839

[B32] Hegde P. S. Chen D. S. (2020). Top 10 Challenges in Cancer Immunotherapy. Immunity 52 (1), 17–35. 10.1016/j.immuni.2019.12.011 31940268

[B34] Hoff P. M. Ansari R. Batist G. Cox J. Kocha W. Kuperminc M. (20012001). Comparison of Oral Capecitabine versus Intravenous Fluorouracil Plus Leucovorin as First-Line Treatment in 605 Patients with Metastatic Colorectal Cancer: Results of a Randomized Phase III Study. Jco 19 (8), 2282–2292. 10.1200/JCO.2001.19.8.2282 11304782

[B35] Jatoi A. Murphy B. R. Foster N. R. Nikcevich D. A. Alberts S. R. Knost J. A. (2006). Oxaliplatin and Capecitabine in Patients with Metastatic Adenocarcinoma of the Esophagus, Gastroesophageal junction and Gastric Cardia: a Phase II Study from the North Central Cancer Treatment Group. Ann. Oncol. 17 (1), 29–34. 10.1093/annonc/mdj063 16303863

[B36] Jensen S. A. Hasbak P. Mortensen J. Sørensen J. B. (2010). Fluorouracil Induces Myocardial Ischemia with Increases of Plasma Brain Natriuretic Peptide and Lactic Acid but without Dysfunction of Left Ventricle. J. Clin. Oncol. 28 (36), 5280–5286. 10.1200/JCO.2009.27.3953 21079148

[B37] Jensen S. A. Sørensen J. B. (2006). Risk Factors and Prevention of Cardiotoxicity Induced by 5-fluorouracil or Capecitabine. Cancer Chemother. Pharmacol. 58 (4), 487–493. 10.1007/s00280-005-0178-1 16418875

[B38] Jin X. Bai Y. Gao L. Wu S. (2019). Incidence of and Risk Factors for Cardiotoxicity after Fluorouracil-Based Chemotherapy in Locally Advanced or Metastatic Gastric Cancer Patients. Cancer Chemother. Pharmacol. 84 (3), 599–607. 10.1007/s00280-019-03888-1 31203389

[B39] Joensuu H. Bono P. Kataja V. Alanko T. Kokko R. Asola R. (2009). Fluorouracil, Epirubicin, and Cyclophosphamide with Either Docetaxel or Vinorelbine, with or without Trastuzumab, as Adjuvant Treatments of Breast Cancer: Final Results of the FinHer Trial. J. Clin. Oncol. 27 (34), 5685–5692. 10.1200/JCO.2008.21.4577 19884557

[B40] Kanduri J. More L. A. Godishala A. Asnani A. (2019). Fluoropyrimidine-Associated Cardiotoxicity. Cardiol. Clin. 37 (4), 399–405. 10.1016/j.ccl.2019.07.004 31587781

[B41] Karakulak U. N. Aladağ E. Maharjan N. Övünç K. (2016). Capecitabine-induced Coronary Artery Vasospasm in a Patient Who Previously Experienced a Similar Episode with Fluorouracil Therapy. Turk Kardiyol Dern Ars 44 (1), 71–74. 10.5543/tkda.2015.36005 26875134

[B42] Kerr R. S. Love S. Segelov E. Johnstone E. Falcon B. Hewett P. (2016). Adjuvant Capecitabine Plus Bevacizumab versus Capecitabine Alone in Patients with Colorectal Cancer (QUASAR 2): an Open-Label, Randomised Phase 3 Trial. Lancet Oncol. 17 (11), 1543–1557. 10.1016/S1470-2045(16)30172-3 27660192

[B43] Khan M. A. Masood N. Husain N. Ahmad B. Aziz T. Naeem A. (2012). A Retrospective Study of Cardiotoxicities Induced by 5-fluouracil (5-FU) and 5-FU Based Chemotherapy Regimens in Pakistani Adult Cancer Patients at Shaukat Khanum Memorial Cancer Hospital & Research Center. J. Pak Med. Assoc. 62 (5), 430–434. 22755303

[B44] Koca D. Salman T. Unek I. T. Oztop I. Ellidokuz H. Eren M. (2011). Clinical and Electrocardiography Changes in Patients Treated with Capecitabine. Chemotherapy 57 (5), 381–387. 10.1159/000331645 21997165

[B45] Koopman M. Antonini N. F. Douma J. Wals J. Honkoop A. H. Erdkamp F. L. (2006). Randomised Study of Sequential versus Combination Chemotherapy with Capecitabine, Irinotecan and Oxaliplatin in Advanced Colorectal Cancer, an Interim Safety Analysis. A Dutch Colorectal Cancer Group (DCCG) Phase III Study. Ann. Oncol. 17 (10), 1523–1528. 10.1093/annonc/mdl179 16873425

[B46] Kosmas C. Kallistratos M. S. Kopterides P. Syrios J. Skopelitis H. Mylonakis N. (2008). Cardiotoxicity of Fluoropyrimidines in Different Schedules of Administration: a Prospective Study. J. Cancer Res. Clin. Oncol. 134 (1), 75–82. 10.1007/s00432-007-0250-9 17636329 PMC12161746

[B47] Koutsoukis A. Ntalianis A. Repasos E. Kastritis E. Dimopoulos M. A. Paraskevaidis I. (2018). Cardio-oncology: A Focus on Cardiotoxicity. Eur. Cardiol. 13 (1), 64–69. 10.15420/ecr.2017:17:2 30310475 PMC6159462

[B48] Kurokawa J. Suzuki T. Furukawa T. (2009). New Aspects for the Treatment of Cardiac Diseases Based on the Diversity of Functional Controls on Cardiac Muscles: Acute Effects of Female Hormones on Cardiac Ion Channels and Cardiac Repolarization. J. Pharmacol. Sci. 109 (3), 334–340. 10.1254/jphs.08r23fm 19270425

[B49] Kwakman J. J. Simkens L. H. Mol L. Kok W. E. Koopman M. Punt C. J. (2017). Incidence of Capecitabine-Related Cardiotoxicity in Different Treatment Schedules of Metastatic Colorectal Cancer: A Retrospective Analysis of the CAIRO Studies of the Dutch Colorectal Cancer Group. Eur. J. Cancer 76, 93–99. 10.1016/j.ejca.2017.02.009 28286287

[B50] Leicher L. W. de Graaf J. C. Coers W. Tascilar M. de Groot J. W. (2017). Tolerability of Capecitabine Monotherapy in Metastatic Colorectal Cancer: A Real-World Study. Drugs R. D 17 (1), 117–124. 10.1007/s40268-016-0154-8 27848234 PMC5318322

[B51] Lestuzzi C. Tartuferi L. Viel E. Buonadonna A. Vaccher E. Berretta M. (2020). Fluoropyrimidine-Associated Cardiotoxicity: Probably Not So Rare as it Seems. Oncologist 25 (8), e1254. 10.1634/theoncologist.2020-0053 32436298 PMC7418346

[B52] Lestuzzi C. Vaccher E. Talamini R. Lleshi A. Meneguzzo N. Viel E. (2014). Effort Myocardial Ischemia during Chemotherapy with 5-fluorouracil: an Underestimated Risk. Ann. Oncol. 25 (5), 1059–1064. 10.1093/annonc/mdu055 24558023

[B53] Li C. Ngorsuraches S. Chou C. Chen L. Qian J. Qian J. (2021). Risk Factors of Fluoropyrimidine Induced Cardiotoxicity Among Cancer Patients: A Systematic Review and Meta-Analysis. Crit. Rev. Oncology/Hematology 162, 103346. 10.1016/j.critrevonc.2021.103346 33930532

[B54] Lordick F. Lorenzen S. Stollfuss J. Vehling-Kaiser U. Kullmann F. Hentrich M. (2005). Phase II Study of Weekly Oxaliplatin Plus Infusional Fluorouracil and Folinic Acid (FUFOX Regimen) as First-Line Treatment in Metastatic Gastric Cancer. Br. J. Cancer 93 (2), 190–194. 10.1038/sj.bjc.6602697 16012522 PMC2361546

[B55] Lotrionte M. Biondi-Zoccai G. Abbate A. Lanzetta G. D'Ascenzo F. Malavasi V. (2013). Review and Meta-Analysis of Incidence and Clinical Predictors of Anthracycline Cardiotoxicity. Am. J. Cardiol. 112 (12), 1980–1984. 10.1016/j.amjcard.2013.08.026 24075281

[B56] Lounsbury P. Elokda A. S. Bunning J. M. Arena R. Gordon E. E. (2017). The Value of Detecting Asymptomatic Signs of Myocardial Ischemia in Patients with Coronary Artery Disease in Outpatient Cardiac Rehabilitation. J. Cardiovasc. Nurs. 32 (3), E1–E9. 10.1097/JCN.0000000000000380 27879618

[B57] Machiels J. P. Sempoux C. Scalliet P. Coche J. C. Humblet Y. Van Cutsem E. (2007). Phase I/II Study of Preoperative Cetuximab, Capecitabine, and External Beam Radiotherapy in Patients with Rectal Cancer. Ann. Oncol. 18 (4), 738–744. 10.1093/annonc/mdl460 17208931

[B58] Mambrini A. Sanguinetti F. Pacetti P. Caudana R. Iacono C. Guglielmi A. (2006). Intra-arterial Infusion of 5-fluorouracil, Leucovorin, Epirubicin and Carboplatin (FLEC Regimen) in Unresectable Pancreatic Cancer: Results of a Ten-Year Experience. In Vivo 20 (6A), 751–755. 10.1007/978-3-642-04346-8_23 17203761

[B59] Martín M. Makhson A. Gligorov J. Lichinitser M. Lluch A. Semiglazov V. (2012). Phase II Study of Bevacizumab in Combination with Trastuzumab and Capecitabine as First-Line Treatment for HER-2-Positive Locally Recurrent or Metastatic Breast Cancer. Oncologist 17 (4), 469–475. 10.1634/theoncologist.2011-0344 22467666 PMC3336828

[B60] Masi G. Loupakis F. Salvatore L. Fornaro L. Cremolini C. Cupini S. (2010). Bevacizumab with FOLFOXIRI (Irinotecan, Oxaliplatin, Fluorouracil, and Folinate) as First-Line Treatment for Metastatic Colorectal Cancer: a Phase 2 Trial. Lancet Oncol. 11 (9), 845–852. 10.1016/S1470-2045(10)70175-3 20702138

[B61] Mayer I. A. Zhao F. Arteaga C. L. Symmans W. F. Park B. H. Burnette B. L. (2021). Randomized Phase III Postoperative Trial of Platinum-Based Chemotherapy versus Capecitabine in Patients with Residual Triple-Negative Breast Cancer Following Neoadjuvant Chemotherapy: ECOG-ACRIN EA1131. J. Clin. Oncol. 39 (23), 2539–2551. 10.1200/JCO.21.00976 34092112 PMC8577688

[B62] Mayer R. J. Van Cutsem E. Falcone A. Yoshino T. Garcia-Carbonero R. Mizunuma N. (2015). Randomized Trial of TAS-102 for Refractory Metastatic Colorectal Cancer. N. Engl. J. Med. 372 (20), 1909–1919. 10.1056/nejmoa1414325 25970050

[B63] Meydan N. Kundak I. Yavuzsen T. Oztop I. Barutca S. Yilmaz U. (2005). Cardiotoxicity of de Gramont's Regimen: Incidence, Clinical Characteristics and Long-term Follow-up. Jpn. J. Clin. Oncol. 35 (5), 265–270. 10.1093/jjco/hyi071 15855175

[B64] Michalaki V. Fotiou S. Gennatas S. Gennatas C. (2010). Trastuzumab Plus Capecitabine and Docetaxel as First-Line Therapy for HER2-Positive Metastatic Breast Cancer: Phase II Results. Anticancer Res. 30 (7), 3051–3054. 10.1200/jco.2009.27.15_suppl.1111 20683054

[B65] Mohammed R. Sallam N. El-Abhar H. (2018). P47885-Fluorouracil Cardiotoxicity: the Role of Oxidative Stress, Apoptosis, Inflammation and Endothelial Dysfuction. Eur. Heart J. 39, 1003–1004. 10.1093/eurheartj/ehy563.P4788

[B66] More L. A. Lane S. Asnani A. (2021). 5-FU Cardiotoxicity: Vasospasm, Myocarditis, and Sudden Death. Curr. Cardiol. Rep. 23 (3), 17. 10.1007/s11886-021-01441-2 33537861

[B67] Ng M. Cunningham D. Norman A. R. (2005). The Frequency and Pattern of Cardiotoxicity Observed with Capecitabine Used in Conjunction with Oxaliplatin in Patients Treated for Advanced Colorectal Cancer (CRC). Eur. J. Cancer 41 (11), 1542–1546. 10.1016/j.ejca.2005.03.027 15978800

[B68] Nhlbi Study Quality Assessment Tools (2020). National Institutes of Health (NIH), National Heart, Lung, and Blood Institute (NHLBI). Bethesda, MD, USA. Available at: https://www.nhlbi.nih.gov/healthtopics/study-quality-assessment-tools (accessed on February 10, 2020).

[B69] O'Connell M. J. Colangelo L. H. Beart R. W. Petrelli N. J. Allegra C. J. Sharif S. (2014). Capecitabine and Oxaliplatin in the Preoperative Multimodality Treatment of Rectal Cancer: Surgical End Points from National Surgical Adjuvant Breast and Bowel Project Trial R-04. J. Clin. Oncol. 32 (18), 1927–1934. 10.1200/JCO.2013.53.7753 24799484 PMC4050205

[B70] Okines A. F. Langley R. E. Thompson L. C. Stenning S. P. Stevenson L. Falk S. (2013). Bevacizumab with Peri-Operative Epirubicin, Cisplatin and Capecitabine (ECX) in Localised Gastro-Oesophageal Adenocarcinoma: a Safety Report. Ann. Oncol. 24 (3), 702–709. 10.1093/annonc/mds533 23108952

[B71] Pai V. B. Nahata M. C. (2000). Cardiotoxicity of Chemotherapeutic Agents: Incidence, Treatment and Prevention. Drug Saf. 22 (4), 263–302. 10.2165/00002018-200022040-00002 10789823

[B72] Peiffert D. Giovannini M. Ducreux M. Michel P. François E. Lemanski C. (2001). High-dose Radiation Therapy and Neoadjuvant Plus Concomitant Chemotherapy with 5-fluorouracil and Cisplatin in Patients with Locally Advanced Squamous-Cell Anal Canal Cancer: Final Results of a Phase II Study. Ann. Oncol. 12 (3), 397–404. 10.1023/A:1011107105538 11332154

[B73] Peng J. Dong C. Wang C. Li W. Yu H. Zhang M. (2018). Cardiotoxicity of 5-fluorouracil and Capecitabine in Chinese Patients: a Prospective Study. Cancer Commun. (Lond) 38 (1), 22. 10.1186/s40880-018-0292-1 29764506 PMC5953402

[B74] Petrini I. Lencioni M. Ricasoli M. Iannopollo M. Orlandini C. Oliveri F. (2012). Phase II Trial of Sorafenib in Combination with 5-fluorouracil Infusion in Advanced Hepatocellular Carcinoma. Cancer Chemother. Pharmacol. 69 (3), 773–780. 10.1007/s00280-011-1753-2 22033636

[B75] Polk A. Shahmarvand N. Vistisen K. Vaage-Nilsen M. Larsen F. O. Schou M. (2016). Incidence and Risk Factors for Capecitabine-Induced Symptomatic Cardiotoxicity: a Retrospective Study of 452 Consecutive Patients with Metastatic Breast Cancer. BMJ Open 6, e012798. 10.1136/bmjopen-2016-012798 PMC5073470 27798021

[B76] Primrose J. N. Fox R. P. Palmer D. H. Malik H. Z. Prasad R. Mirza D. (2019). Capecitabine Compared with Observation in Resected Biliary Tract Cancer (BILCAP): a Randomised, Controlled, Multicentre, Phase 3 Study. Lancet Oncol. 20 (5), 663–673. 10.1016/S1470-2045(18)30915-X 30922733

[B77] Raber I. Warack S. Kanduri J. Pribish A. Godishala A. Abovich A. (2019). Fluoropyrimidine-Associated Cardiotoxicity: A Retrospective Case-Control Study. Oncologist 25 (3), E606–E609. 10.1634/theoncologist.2019-0762 32162823 PMC7066698

[B78] Sara J. D. Kaur J. Khodadadi R. Rehman M. Lobo R. Chakrabarti S. (2018). 5-fluorouracil and Cardiotoxicity: a Review. Ther. Adv. Med. Oncol. 10, 1758835918780140. 10.1177/1758835918780140 29977352 PMC6024329

[B79] Saunders S. Anwar M. (2019). Capecitabine-induced Myopericarditis - A Case Report and Review of Literature. J. Oncol. Pharm. Pract. 25 (4), 1006–1010. 10.1177/1078155218774871 29783917

[B80] Sedhom D. Sedhom R. Khan W. (2017). A Rare Case of Acute Coronary Syndrome Induced by Oral Capecitabine. Am. J. Resp. Crit. Care 195. 10.1016/S0735-1097(21)03914-0

[B81] Skof E. Rebersek M. Hlebanja Z. Ocvirk J. (2009). Capecitabine Plus Irinotecan (XELIRI Regimen) Compared to 5-FU/LV Plus Irinotecan (FOLFIRI Regimen) as Neoadjuvant Treatment for Patients with Unresectable Liver-Only Metastases of Metastatic Colorectal Cancer: a Randomised Prospective Phase II Trial. BMC Cancer 9 (120), 120. 10.1186/1471-2407-9-120 19386096 PMC2678276

[B82] Smith L. A. Cornelius V. R. Plummer C. J. Levitt G. Verrill M. Canney P. (2010). Cardiotoxicity of Anthracycline Agents for the Treatment of Cancer: Systematic Review and Meta-Analysis of Randomised Controlled Trials. BMC Cancer 10, 337. 10.1186/1471-2407-10-337 20587042 PMC2907344

[B83] Tonyali O. Benekli M. Berk V. Coskun U. Ozkan M. Yildiz R. (2013). Efficacy and Toxicity of Trastuzumab and Paclitaxel Plus Capecitabine in the First-Line Treatment of HER2-Positive Metastatic Breast Cancer. J. Cancer Res. Clin. Oncol. 139 (6), 981–986. 10.1007/s00432-013-1409-1 23463098 PMC11824636

[B84] Tsavaris N. Kosmas C. Vadiaka M. Efremidis M. Zinelis A. Beldecos D. (2002). Cardiotoxicity Following Different Doses and Schedules of 5-fluorouracil Administration for Malignancy -- a Survey of 427 Patients. Med. Sci. Monit. 8 (6), PI51–7. 10.12659/MSM.936523 12070449

[B85] Turan T. Agac M. T. Aykan A. Ç. Kul S. Akyüz A. R. Gökdeniz T. (2017). Usefulness of Heart-type Fatty Acid-Binding Protein and Myocardial Performance Index for Early Detection of 5-Fluorouracil Cardiotoxicity. Angiology 68 (1), 52–58. 10.1177/0003319716637516 26980771

[B86] Vaishampayan U. N. Ben-Josef E. Philip P. A. Vaitkevicius V. K. Du W. Levin K. J. (2002). A Single-Institution Experience with Concurrent Capecitabine and Radiation Therapy in Gastrointestinal Malignancies. Int. J. Radiat. Oncol. Biol. Phys. 53, 675–679. (PII S0360-9(02)02772-43). 10.1016/S0360-3016(02)02772-4 12062611

[B87] Van Cutsem E. Hoff P. M. Blum J. L. Abt M. Osterwalder B. (2002). Incidence of Cardiotoxicity with the Oral Fluoropyrimidine Capecitabine Is Typical of that Reported with 5-fluorouracil. Ann. Oncol. 13, 484–485. 10.1093/annonc/mdf108 11996484

[B88] Wacker A. Lersch C. Scherpinski U. Reindl L. Seyfarth M. (2003). High Incidence of Angina Pectoris in Patients Treated with 5-fluorouracil. A Planned Surveillance Study with 102 Patients. ONCOLOGY 65 (2), 108–112. 10.1159/000072334 12931015

[B89] Winther S. B. Zubcevic K. Qvortrup C. Vestermark L. W. Jensen H. A. Krogh M. (2016). Experience with S-1 in Older Caucasian Patients with Metastatic Colorectal Cancer (mCRC): Findings from an Observational Chart Review. Acta Oncol. 55 (7), 881–885. 10.3109/0284186X.2016.1161825 27181284

[B90] Yamamoto D. Iwase S. Kitamura K. Odagiri H. Yamamoto C. Nagumo Y. (2008). A Phase II Study of Trastuzumab and Capecitabine for Patients with HER2-Overexpressing Metastatic Breast Cancer: Japan Breast Cancer Research Network (JBCRN) 00 Trial. Cancer Chemother. Pharmacol. 61 (3), 509–514. 10.1007/s00280-007-0497-5 17516068

[B91] Yerushalmi R. Idelevich E. Dror Y. Stemmer S. M. Figer A. Sulkes A. (2006). Preoperative Chemoradiation in Rectal Cancer: Retrospective Comparison between Capecitabine and Continuous Infusion of 5-fluorouracil. J. Surg. Oncol. 93 (7), 529–533. 10.1002/jso.20503 16705722

[B92] Yilmaz U. Oztop I. Ciloglu A. Okan T. Tekin U. Yaren A. (2007). 5-Fluorouracil Increases the Number and Complexity of Premature Complexes in the Heart: a Prospective Study Using Ambulatory ECG Monitoring. Int. J. Clin. Pract. 61 (5), 795–801. 10.1111/j.1742-1241.2007.01323.x 17493091

[B93] Zafar A. Drobni Z. D. Mosarla R. Alvi R. M. Lei M. Lou U. Y. (2021). The Incidence, Risk Factors, and Outcomes with 5-Fluorouracil-Associated Coronary Vasospasm. JACC CardioOncol 3 (1), 101–109. 10.1016/j.jaccao.2020.12.005 33817666 PMC8018593

[B94] Zhang P. Sun T. Zhang Q. Yuan Z. Jiang Z. Wang X. J. (2017). Utidelone Plus Capecitabine versus Capecitabine Alone for Heavily Pretreated Metastatic Breast Cancer Refractory to Anthracyclines and Taxanes: a Multicentre, Open-Label, Superiority, Phase 3, Randomised Controlled Trial. Lancet Oncol. 18 (3), 371–383. 10.1016/S1470-2045(17)30088-8 28209298

