# Supplementary material for: Treatment-Related Coronary Disorders of Fluoropyrimidine Administration: A Systematic Review and Meta-Analysis
Source: Front Pharmacol. 2022 May 13;13:885699. doi: 10.3389/fphar.2022.885699 (PMC9140752; doi:10.3389/fphar.2022.885699)
Supplement: Supplementary file 1 [file Table1.docx]

| 1. **The National Institutes of Health (NIH) quality assessment tool for case-series study** | | | |
| --- | --- | --- | --- |
| **Major Components** | **Response Options** | | |
| 1. Was the study question or objective clearly stated? | Yes | No | Cannot Determine/ Not Applicable/ Not Reported |
| 2. Was the study population clearly and fully described, including a case definition? | Yes | No | Cannot Determine/ Not Applicable/ Not Reported |
| 3. Were the cases consecutive? | Yes | No | Cannot Determine/ Not Applicable/ Not Reported |
| 4. Were the subjects comparable? | Yes | No | Cannot Determine/ Not Applicable/ Not Reported |
| 5. Was the intervention clearly described? | Yes | No | Cannot Determine/ Not Applicable/ Not Reported |
| 6. Were the outcome measures clearly defined, valid, reliable, and implemented consistently across all study participants? | Yes | No | Cannot Determine/ Not Applicable/ Not Reported |
| 7. Was the length of follow-up adequate? | Yes | No | Cannot Determine/ Not Applicable/ Not Reported |
| 8. Were the statistical methods well-described? | Yes | No | Cannot Determine/ Not Applicable/ Not Reported |
| 9. Were the results well-described? | Yes | No | Cannot Determine/ Not Applicable/ Not Reported |
| **Quality Rating** | **Good** | **Fair** | **Poor** |
| Additional Comments (If Poor, please state why): | | | |

Supplementary Material

# Supplementary Table 1 The National Institutes of Health (NIH) quality assessment tools

**Website:** https://www.nhlbi.nih.gov/health-topics/study-quality-assessment-tools

| **2. The National Institutes of Health (NIH) quality assessment tool of controlled intervention study** | | | |
| --- | --- | --- | --- |
| **Major Components** | **Response options** | | |
| 1. Was the study described as randomized, a randomized trial, a randomized clinical trial, or an RCT? | Yes | No | Cannot Determine/ Not Applicable/ Not Reported |
| 2. Was the method of randomization adequate (i.e., use of randomly generated assignment)? | Yes | No | Cannot Determine/ Not Applicable/ Not Reported |
| 3. Was the treatment allocation concealed (so that assignments could not be predicted)? | Yes | No | Cannot Determine/ Not Applicable/ Not Reported |
| 4. Were study participants and providers blinded to treatment group assignment? | Yes | No | Cannot Determine/ Not Applicable/ Not Reported |
| 5. Were the people assessing the outcomes blinded to the participants' group assignments? | Yes | No | Cannot Determine/ Not Applicable/ Not Reported |
| 6. Were the groups similar at baseline on important characteristics that could affect outcomes (e.g., demographics, risk factors, co-morbid conditions)? | Yes | No | Cannot Determine/ Not Applicable/ Not Reported |
| 7. Was the overall drop-out rate from the study at endpoint 20% or lower of the number allocated to treatment? | Yes | No | Cannot Determine/ Not Applicable/ Not Reported |
| 8. Was the differential drop-out rate (between treatment groups) at endpoint 15 percentage points or lower? | Yes | No | Cannot Determine/ Not Applicable/ Not Reported |
| 9. Was there high adherence to the intervention protocols for each treatment group? | Yes | No | Cannot Determine/ Not Applicable/ Not Reported |
| 10. Were other interventions avoided or similar in the groups (e.g., similar background treatments)? | Yes | No | Cannot Determine/ Not Applicable/ Not Reported |
| 11. Were outcomes assessed using valid and reliable measures, implemented consistently across all study participants? | Yes | No | Cannot Determine/ Not Applicable/ Not Reported |
| 12. Did the authors report that the sample size was sufficiently large to be able to detect a difference in the main outcome between groups with at least 80% power? | Yes | No | Cannot Determine/ Not Applicable/ Not Reported |
| 13. Were outcomes reported or subgroups analyzed prespecified (i.e., identified before analyses were conducted)? | Yes | No | Cannot Determine/ Not Applicable/ Not Reported |
| 14. Were all randomized participants analyzed in the group to which they were originally assigned, i.e., did they use an intention-to-treat analysis? | Yes | No | Cannot Determine/ Not Applicable/ Not Reported |
| **Quality Rating** | Good | Fair | Poor |
| Additional Comments (If Poor, please state why): | | | |
